# How Can General Self-Efficacy Facilitate Undergraduates’ Employability? A Multiple Mediation Model

**DOI:** 10.3390/bs15040514

**Published:** 2025-04-11

**Authors:** Jiajia Tan, Jinglin Li, Xinfa Yi

**Affiliations:** 1 Faculty of Education, Shaanxi Normal University, Xi’an 710062, China; jiajiatan@snnu.edu.cn; 2 College of Traffic and Transportation, Chongqing Jiaotong University, Chongqing 400074, China; 3 Key Laboratory of Modern Teaching Technology, Ministry of Education of China, Shaanxi Normal University, 199# South Chang’an Road, Xi’an 710062, China; lijinglin@snnu.edu.cn

**Keywords:** general self-efficacy, achievement motivation, occupational aspiration, undergraduates’ employability

## Abstract

Self-efficacy is a key cognitive factor affecting undergraduates’ employability, but existing studies have not sufficiently explored the underlying mechanisms of how self-efficacy influences employability, not to mention proposing a comprehensive model to clarify these pathways. Based on social cognitive career theory (SCCT), this study developed an empirical model to explore how achievement motivation and occupational aspiration mediate the relationship between general self-efficacy and employability. A survey was conducted with 454 undergraduates from five regional universities with industrial features in mainland China. The results showed that (1) there was a significant positive correlation between the four variables of general self-efficacy, achievement motivation, occupational aspiration and undergraduates’ employability; (2) general self-efficacy had a significant positive effect on undergraduates’ employability; and (3) general self-efficacy could affect undergraduates’ employability through three indirect paths, namely, the independent mediation of achievement motivation, occupational aspiration and the chain mediation of achievement motivation and occupational aspiration. This study provides insights into how self-efficacy enhances employability, suggesting that educational interventions focusing on boosting students’ self-efficacy and fostering achievement motivation and occupational aspiration are effective strategies for improving employability.

## 1. Introduction

Employment is the foundation of people’s welfare. The employability of undergraduates is essential for economic development, social stability, and national development. However, the employability of undergraduates is facing unprecedented challenges in the context of global labor market transformations. Economic volatility, government employment policies, applicants’ interview performance, competence and self-cognition have compounded employment uncertainties (Su & Zhang, 2015). Concurrently, rapid technological digitalization—encompassing automation, robotics, and AI-driven productivity tools—has redefined skill demands across industries (Jung et al., 2024), further threatening undergraduates’ self-efficacy in employment. According to McKinsey’s 2021 report, up to one-third of the world’s job and skill changes may occur in China by 2030, and as many as 220 million Chinese workers may change careers due to the impact of automation technology or robotics (McKinsey & Company, 2021). The data released by the National Bureau of Statistics indicated that in May 2023, the unemployment rate for the labor force aged 16 to 24 in China was 20.8% (Lai & He, 2023). However, the number of Chinese college graduates in 2025 is still growing, with an increase of 430,000 over the previous year (Ministry of Education, 2024), further exacerbating the employment challenges. These systematic challenges are particularly acute for students from regional universities with industrial features. It is stated that due to the limited education resources and narrow employment opportunities, the employability of technology undergraduates from regional universities is significantly deficient (Y. X. Ma & Liang, 2016). Thus, understanding the underlying mechanisms that influence employability is crucial for addressing the employment challenges faced by those graduates.

Employability is the crucial capability for undergraduates to achieve economic independence, personal growth and social adaptation, which has long been a high concern of graduates, higher education institutions and society. However, the notion of employability is hard to define. Employability is a multidimensional concept that encompasses both subjective and objective elements. In the context of higher education, the objective notion of employability involves acquiring a set of domain knowledge (e.g., technical knowledge), soft skills (e.g., interpersonal aptitude) and other personal characteristics (e.g., conscientiousness) that are highly valued by employers (Bonnard, 2020). In the subjective aspect, employability is defined as an individual’s perceived capacity to get, maintain and advance in employment (Rothwell & Arnold, 2007). Perceived employability increases the probability of successful employment because perceptions motivate behaviors. Individuals with high perceived employability are more competent in identifying career opportunities, which will drive them to manage their careers more proactively and successfully. Moreover, employability is typically defined in terms of objective indicators or observable abilities that can be directly measured. However, it is essential to regard employability not just through employment outcomes (objectively) but also through the individual’s personal experience (subjectively) (Neroorkar, 2022). Hence, the aim of this study is to investigate undergraduates’ perceived employability. Numerous factors may influence employability, including internal and external elements, such as professional ability, psychological endurance, employment policies and labor market conditions (Su & Zhang, 2015). Existing research has mainly focused on external factors influencing the employability of undergraduates, while little attention has been given to the role of general self-efficacy within the broader framework of an individual’s psychological capital. However, in contrast with external factors, internal psychological factors, such as general self-efficacy, are often more influential in shaping employability (George et al., 2022). Studies have demonstrated that general self-efficacy, as an individual trait, is closely associated with personal job performance, and this association remains relatively stable regardless of labor market conditions (Judge & Bono, 2001), which suggests that enhancing general self-efficacy may be a reliable approach to facilitating undergraduates’ employability.

General self-efficacy refers to an individual’s overall degree of confidence in coping with challenges in various environments (Schwarzer et al., 1997). Social cognitive career theory posits that self-efficacy is the core cognitive variable driving individuals’ job-seeking behaviors, thereby increasing job opportunities and success rates of employment. Some studies revealed that self-efficacy directly and positively predicts students’ employability (Mason et al., 2022), while some studies confirmed that self-efficacy affects employability through various mediators, such as achievement motivation (Remedios & Sewell, 2024), occupational aspiration (Gbadamosi et al., 2015) and so on. Still, other research found that the impact of self-efficacy on personal performance is diverse, which could be positive, negative or have no significant impact, and higher self-efficacy does not predict individual performance in all situations (Vancouver et al., 2001). Most of these studies mainly focus on discussing the impact of one or two psychological factors on undergraduates’ employability, offering valuable insights into the relationship between general self-efficacy and undergraduates’ employability. However, several gaps remain in the literature, including the absence of a comprehensive theoretical framework and explanatory model. Additionally, existing research findings are often inconsistent, highlighting the need for further investigation.

### 1.1. General Self-Efficacy and Employability

According to social cognitive career theory (SCCT), an individual’s career development path is the result of the interaction of various career elements within the “person–behavior–environment” interactive system, among which self-efficacy, outcome expectations and choice goals are the three core cognitive variables that drive an individual’s learning and job hunting (Lent et al., 1994).

Self-efficacy refers to an individual’s confidence in his ability to complete a certain task or job, which is a subjective judgment of his own capability and behavioral performance (Bandura, 1977). When individuals believe they are capable of handling a certain job, they are more likely to take actions to pursue that job. Self-efficacy can be categorized into general self-efficacy and specific self-efficacy. Compared with specific self-efficacy, which is characterized by task or domain specificity, general self-efficacy involves an individual’s comprehensive evaluation, focuses more on the individual’s overall capabilities and has a greater and more profound impact on the individual’s overall confidence level, enabling the individual to be more confident when dealing with tasks in unfamiliar fields. Considering that undergraduates’ employability reflects their perceived overall competencies, this study takes general self-efficacy as the independent variable to explore its impact on undergraduates’ employability. Although previous research has not reached a consensus on the influence of general self-efficacy on undergraduates’ employability, more evidence supports that self-efficacy, as a personal trait, positively predicts undergraduates’ employability, and this impact is consistent across different cultures (Vancouver et al., 2001; Zhao et al., 2021). Based on this, the following hypothesis is proposed:

**H1:**  *General self-efficacy positively predicts undergraduates’ employability.*

### 1.2. The Mediating Role of Achievement Motivation

Achievement motivation refers to the psychological drive for individuals to pursue excellence and make relentless efforts, involving two distinct tendencies: the pursuit of success and the avoidance of failure (Atkinson, 1957). As a key component of self-regulatory strategy, achievement motivation involves the behavior of starting and persisting with a task based on self-efficacy and internal motivation. According to social cognitive career theory, individuals with high self-efficacy hold more positive outcome expectations for job-seeking, including the anticipation of achieving success and obtaining self-recognized outcomes (Lent et al., 1994), which shares a similar connotation with achievement motivation. Empirical research also has demonstrated that general self-efficacy is a significant antecedent factor in enhancing undergraduates’ achievement motivation (Kim & Shin, 2021). Moreover, achievement motivation is an important driving force for job-seeking actions, which contributes to improving employability (Li et al., 2022). Achievement motivation is closely related to situational factors. Different achievement situations will activate different achievement motivations—for example, the motivation to achieve success or avoid failure. However, whether the motivation to achieve success or avoid failure is activated, the motives have three essential functions: choosing, orienting and energizing behavior (McClelland, 1987), which provides a clear direction for individuals to engage in job search activities. Therefore, when job-search outcomes diverge from expectations, individuals can regulate their achievement motivation to clarify the direction of their endeavors and increase their employability. Accordingly, the following hypothesis is proposed:

**H2:**  *Achievement motivation mediates the relationship between general self-efficacy and undergraduates’ employability.*

### 1.3. Mediating Role of Occupational Aspirations

Occupational aspirations refer to an individual’s desired career goals or job wishes given ideal circumstances (Lent et al., 1994), which are future-oriented and in relation to specific beliefs about one’s future career trajectory. The definition is consistent, in connotation, with the choice goal. The social cognitive career theory holds that a choice goal is an individual’s intention to choose or adhere to a certain action and is a key factor in predicting subsequent job-seeking actions. The more specific and feasible the choice goals or occupational aspirations are, the more likely individuals are to convert them into employment actions. The Job Demands-Resources model indicates that occupational aspirations serve as a catalyst for employees to achieve better performance (Bakker & Demerouti, 2007). Additionally, individuals or teams with higher levels of self-efficacy tend to set higher goals for themselves and, therefore, possess higher levels of aspiration. Based on this, the following hypothesis is proposed:

**H3:**  *Occupational aspirations mediate the relationship between general self-efficacy and undergraduates’ employability.*

### 1.4. The Chain Mediating Role of Achievement Motivation and Occupational Aspirations

Previous studies suggest that students’ general self-efficacy impacts employability, with this relationship potentially mediated by regulatory strategies (e.g., achievement motivation) or career beliefs (e.g., occupational aspiration). However, few empirical studies investigate whether achievement motivation and occupational aspiration work together as chain mediators in the relationship between general self-efficacy and employability.

Occupational aspiration belongs to idealistic aspiration, which indicates the future occupation or career goal that a person desires to obtain without considering the realistic constraints, while achievement motivation represents realistic aspiration, which is also called expectation (Miyamoto et al., 2020), concerning what a person believes he or she can achieve under a specific situation. Both achievement motivation and occupational aspiration might be incentives for individuals to engage in employment activities, but there is always a discrepancy between idealistic aspiration (occupational aspiration) and expectation (achievement motivation). To reduce the aspiration–expectation discrepancies, students can regulate their expectations or aspirations, not as a compromise but as a strategy to facilitate career development in a more realistic or accessible way.

Furthermore, achievement motivation is an important antecedent of occupational aspiration. According to the situated expectancy–value theory (SEVT), individuals’ expectations of success and their subjective assessment of task values are the most proximal determinants of occupational choices (Strohmeier et al., 2025). Achievement motivation, as crucial feedback on students’ abilities, directs them toward achievable and realistic career trajectories. Students with higher achievement motivation often hold higher levels of occupational aspirations, demonstrate greater psychological resilience and perseverance, and tend to integrate their interests and occupational aspirations into their learning activities, thereby enhancing their employability.

In addition, self-efficacy is an important antecedent variable that influences achievement motivation. Individuals with higher self-efficacy often exhibit stronger achievement motivation, prefer to set higher goals, and challenge more difficult tasks. Based on this, the following hypothesis is proposed:

**H4:**  *Achievement motivation and occupational aspirations play a chain mediating role between general self-efficacy and the employability of undergraduates.*

In summary, existing research has consistently demonstrated close associations among general self-efficacy, achievement motivation, occupational aspiration and employability, and all of them have significant predictive effects on employability. However, the majority of prior studies have primarily focused on the relationship between a single psychological factor and undergraduates’ employability, often overlooking a systematic exploration of the interrelationships among multiple psychological factors influencing undergraduates’ employability and their underlying mechanisms. Few studies have combined three or more psychological factors to systematically discuss the impact mechanisms on undergraduates’ employability, and fewer have employed a robust model to reveal their causal pathways (Y. Ma & Bennett, 2024; Brown & Lent, 2023). Based on this, the present study aims to construct a hypothesized model with general self-efficacy as the independent variable, achievement motivation and occupational aspirations as the mediating variables and employability as the dependent variable to examine the mechanisms through which general self-efficacy influences employability.

## 2. Materials and Methods

### 2.1. Participants and Sampling

The convenience sampling method is employed to recruit participants from five regional universities with industrial features in Chongqing, China, including Chongqing Jiaotong University, Chongqing University of Posts and Telecommunications, Chongqing Technology and Business University, Chongqing University of Science and Technology and Chongqing University of Technology. All five regional universities have a similar academic level, and none of them are “Double First-Class” universities in China, with their national university rankings ranging from 152 to 237. Ethical review approval for this study is granted by the Ethics Committee of the Key Laboratory of Modern Teaching Technology, Ministry of Education, Shaanxi Normal University, under approval number L20231130-01. The inclusion criteria for samples are (1) participants are full-time undergraduates varying from freshmen to seniors, (2) participants voluntarily consent to take part in the study, (3) participants are capable of comprehending the content of the questionnaires and completing them independently and (4) the average time for completing the brief demographic survey and related questionnaires is more than three minutes. The exclusion criteria for samples are (1) participants completing questionnaires with patterned responses (for example, repeatedly selecting the same option) and (2) participants who are part-time students or no longer enrolled. Initially, the researchers contacted teachers via cellphone to ensure they were willing to invite their students to participate in the study. After obtaining informed consent from the students, participants were invited to complete questionnaires through the Wenjuanxing (http://www.wjx.cn) platform from 11 December to 20 December 2023. After excluding 20 invalid questionnaires, 454 valid questionnaires remain, representing an effective rate of 95.78%. Among the participants, there are 305 males (67.18%) and 149 females (32.82%); 130 freshmen (28.63%), 100 sophomores (22.03%), 86 juniors (18.94%) and 138 seniors (30.40%); 67 participants (14.76%) from non-STEM majors and 387 participants (85.24%) from STEM majors; 120 participants (26.43%) from urban areas, 113 participants (24.89%) from county towns and 221 participants (48.68%) from rural areas.

### 2.2. Measurement of Variables

#### 2.2.1. General Self-Efficacy

The General Self-Efficacy Scale, revised by C. K. Wang et al. (2001), was adopted. It consists of 10 items and employs a Likert 4-point rating scale (1 = “completely incorrect”, 4 = “completely correct”), with higher scores indicating higher levels of general self-efficacy. The Cronbach’s α coefficient for this scale in the present study was 0.919.

#### 2.2.2. Achievement Motivation

The Achievement Motivation Scale revised by Ye and Hagtvet (1992) was adopted. It consists of 30 items covering two dimensions: the pursuit of success and the avoidance of failure. A Likert 5-point rating scale is used (1 = “completely inconsistent”, 5 = “completely consistent”). The higher the score, the stronger the individual’s achievement motivation. In this study, the Cronbach’s α coefficient of the total scale was 0.931, and the coefficients of each dimension were 0.904 and 0.912, respectively.

#### 2.2.3. Occupational Aspirations

The questionnaire on Undergraduates’ Occupational Aspirations, developed by L. J. Wang (2008), was employed. It consists of 25 items across six dimensions: salary, interpersonal relationships, challenging the future, contributing to society, working environment and career prospects. The questionnaire uses a Likert 5-point scale (1 = “extremely unimportant”, 5 = “extremely important”), where higher scores indicate higher levels of occupational aspirations among undergraduates. In this study, the Cronbach’s α coefficient for the overall questionnaire was 0.932, with coefficients of 0.858, 0.837, 0.789, 0.865, 0.716 and 0.666 for each dimension, respectively.

#### 2.2.4. Undergraduates’ Employability

The Undergraduates’ Employability Questionnaire, developed by Zhong (2016), was employed, consisting of 17 items across five dimensions: professional competence; communication competence; collaboration and execution competence; coordination and adaptability competence; and thinking and forecasting competence. A 5-point Likert scale is used for scoring (1 = “completely disagree”, 5 = “completely agree”), with higher scores indicating stronger employability among undergraduates. In this study, Cronbach’s α coefficient for the total questionnaire was 0.946, and the coefficients for each dimension were 0.893, 0.876, 0.900, 0.843 and 0.706, respectively.

### 2.3. Data Processing

The data were analyzed using SPSS 26.0 for the common method bias test, independent sample *t*-tests, one-way ANOVA, descriptive statistics and correlation analysis. The chain mediation model was defined using Model 6 in the macro program PROCESS 3.3 to test the chain mediation effect and to compare the indirect effects of different pathways.

## 3. Results

### 3.1. Common Method Bias Test

In this study, four measurement instruments were used to assess the same participants, and all data were sourced from self-reports. To mitigate common method bias, the anonymity, authenticity and confidentiality of the assessment were emphasized in the instructions before the test began. During the data processing stage, Harman’s single-factor test was used to conduct an exploratory factor analysis on all items from the scales. The results yielded 13 factors with eigenvalues greater than 1, and the variance explained by the largest common factor was 23.326%, which is significantly below the threshold of 40%. Therefore, it is considered that this study does not have notable common method bias issues.

### 3.2. Differences in Demographic Characteristics Among Undergraduates

Participants were categorized into groups based on demographic variables (gender, major, grade and origin). Independent sample *t*-tests and one-way ANOVA were conducted to analyze differences in general self-efficacy, achievement motivation, occupational aspiration and employability across groups. The results are presented in Table 1.

The results showed that undergraduates from different majors exhibited significant differences in general self-efficacy (*t* = −2.63, *p* < 0.01) and employability (*t* = −2.67, *p* < 0.01). Specifically, students with STEM majors demonstrated significantly higher levels of general self-efficacy and employability than those with non-STEM majors. Undergraduates from different origins also presented significant differences in employability (*F* = 3.07, *p* < 0.05). Further post hoc tests indicated that students from urban areas possessed higher employability than those from county towns and rural areas, while no significant difference was detected between students from county towns and rural areas. Apart from that, no significant differences were observed in the research variables across gender or grade. Consequently, major and origin were included as covariates in subsequent analyses to mitigate potential confounding effects on the dependent variable.

### 3.3. Descriptive Statistics and Correlation Analysis

Descriptive statistics and correlation analysis were conducted for all research variables, with the results presented in Table 2. General self-efficacy, achievement motivation, occupational aspirations and employability all showed significant positive correlations with each other.

### 3.4. Chain Mediation Test of Achievement Motivation and Occupational Aspirations

The chain mediation effect was tested using Model 6 in the macro program PROCESS. General self-efficacy was taken as the independent variable, employability as the dependent variable, and achievement motivation and occupational aspirations as the chain mediation variables. Major and origin were included as covariates in the regression equation. The sequential test method and Bootstrap method (with 5000 bootstrap samples) were employed to test the hypothesized model for analyzing the mediating effects.

The results indicated (see Table 3) that all path coefficients were significant, and the variance explained by each model was significant (*F* = 2.961, 10.730, 17.453, *p* < 0.001), with all established model fit values meeting the criteria. General self-efficacy significantly and positively predicted achievement motivation (β = 0.225, *p* < 0.001), occupational aspirations (β = 0.097, *p* < 0.05) and employability (β = 0.279, *p* < 0.001). Achievement motivation significantly and positively predicted occupational aspirations (β = 0.484, *p* < 0.001) and employability (β = 0.100, *p* < 0.05). Additionally, occupational aspirations significantly and positively predicted employability (β = 0.364, *p* < 0.001).

The results of the mediation effect test indicated (see Table 4) that achievement motivation and occupational aspiration played a partial mediating role between general self-efficacy and employability, with a mediation effect value of 0.097, accounting for 25.798% of the total effect. General self-efficacy influenced employability through three mediation pathways. These pathways did not include zero within the 95% confidence interval, indicating that the mediation effects of all three pathways were significant. The specific pathways were (1) general self-efficacy → achievement motivation → employability, with an effect value of 0.023; (2) general self-efficacy →occupational aspiration → employability, with an effect value of 0.035; and (3) general self-efficacy → achievement motivation → occupational aspiration → employability, with an effect value of 0.040.

**Figure 1:**
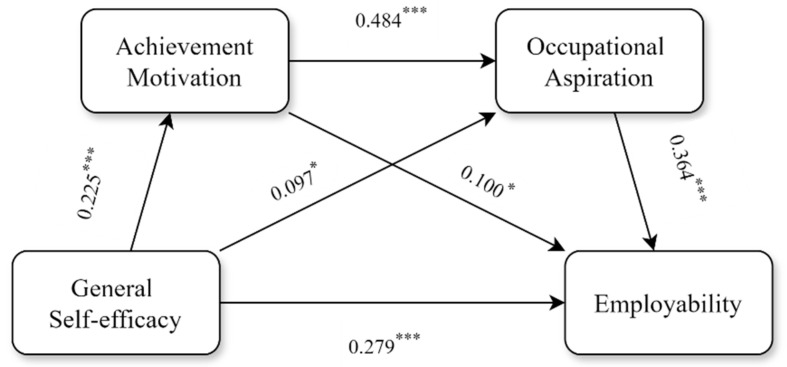
The chain mediating effect of achievement motivation and occupational aspiration between general self-efficacy and employability. Note: * *p* < 0.05, *** *p* < 0.001.

The relationships among the variables are illustrated in Figure 1 based on the above analysis.

In the direct pathway, general self-efficacy had a significant direct effect on undergraduates’ employability, supporting Hypothesis 1.

In the indirect pathways, the total indirect effect was composed of three mediating pathways:

Pathway 1 (general self-efficacy → achievement motivation → employability) demonstrated that achievement motivation had a significant mediating effect between general self-efficacy and undergraduates’ employability, thus validating Hypothesis 2.

Pathway 2 (general self-efficacy → occupational aspiration → employability) indicated that occupational aspiration had a significant mediating effect between general self-efficacy and undergraduates’ employability, thus supporting Hypothesis 3.

Pathway 3 (general self-efficacy → achievement motivation → occupational aspiration → employability) showed that achievement motivation and occupational aspiration played a chain mediating role in the relationship between general self-efficacy and undergraduates’ employability, thereby confirming Hypothesis 4.

## 4. Discussion

### 4.1. The Analysis of Variance in Research Variables Across Demographic Characteristics

Regarding personal characteristics, the study suggests that the role of gender in employability is not significant. However, previous studies from different cultures have confirmed that males perceive higher employability than females (Bennett et al., 2020; Maxwell & Broadbridge, 2014), which is evident across various aspects, such as confidence, career expectations and earnings. The possible explanations for the absence of a gender gap might be attributed to China’s policy support for female employment, and the gender differences might be delayed to being apparent after the transition to the workplace (Maxwell & Broadbridge, 2014). Concerning the year of study, apart from freshmen who may not have a well-developed understanding of employability, there exists a slow rise in employability from sophomores to seniors, which is consistent with human capital theory. That is, the more education individuals receive, the stronger employability they perceive. However, there is no significant difference in undergraduates’ employability across grades. This result could be explained by the fact that undergraduates generally lack work experience, which is directly related to perceived employability. Even among the final-year undergraduates who have participated in internships, the nature of such experiences is significantly different from the employability required in real workplace settings. In terms of students’ origin, urban undergraduates’ employability is significantly higher than that of those from county towns and rural areas. This disparity may be caused by different family socioeconomic statuses. Undergraduates from urban areas are endowed with better social, economic and cultural capital (Bourdieu & Passeron, 1970/1977), thus having higher employability. Finally, the study suggests that undergraduates from STEM majors perceive notably higher employability than those from non-STEM majors. It is argued that, compared with non-STEM majors, STEM majors enjoy greater demand in the labor market, and STEM education is more closely aligned with market needs.

### 4.2. The Mechanism of General Self-Efficacy on Employability

This study reveals that general self-efficacy positively predicts undergraduates’ employability, supporting Hypothesis 1. This finding is consistent with previous research, which highlights the importance of self-efficacy in shaping individuals’ job-seeking behaviors and overall career success (Dacre Pool & Qualter, 2013). General self-efficacy is a crucial factor in determining action choices. When individuals believe in their ability to succeed, they are more likely to engage in goal-directed behaviors, including seeking employment opportunities. Therefore, the higher the level of general self-efficacy that undergraduates hold, the more likely they are to take proactive job-seeking actions. Even when faced with challenges, they will actively use internal and external resources and adopt effective strategies to overcome difficulties. This study extends previous research by specifically examining undergraduates from five regional universities with industrial features in Chongqing, China—a group that faces unique employment challenges. The employment issues faced by this group are unique. These students are future experts in a specific domain, playing a vital role in promoting industry progress and regional economic development. However, due to factors such as limited education resources, undeveloped regional economic conditions and so on, the employment opportunities for these students tend to be limited, accompanied by lower initial salaries and uneven development of employability skills. In addition, the working environment for undergraduates from regional universities with industrial features is not satisfactory. Especially for students majoring in engineering, their working environments are mostly remote, arduous and even dangerous, which inevitably weakens their self-efficacy and leads them to fall into self-doubt as to whether they are competent to handle these challenges. The lack of general self-efficacy negatively impacts their employability, and some undergraduates even resign after employment. The findings of this study underscore the importance of enhancing general self-efficacy among undergraduates, especially those from disadvantaged or specialized academic backgrounds. This research finding provides an important reference for teachers to improve undergraduates’ employability by enhancing their general self-efficacy.

### 4.3. The Mediating Role of Achievement Motivation in the Relationship Between General Self-Efficacy and Employability

The results indicate that achievement motivation significantly mediates the relationship between general self-efficacy and employability, thus validating Hypothesis 2. The result aligns with previous research (Iroegbu, 2015), which emphasizes the critical role of motivation in the employment process. Employability not only represents the perceived ability to get a job but also the perceived ability to convert idealized employment expectations into concrete attainment. However, the employment process is highly dynamic and influenced by various external and internal factors, such as fluctuations in the job market, technological progress, individual skills, career prospects and so on. Consequently, employment outcomes are inherently uncertain. Negative employment experiences, such as failure to obtain a desired job, can undermine individuals’ self-confidence, leading to feelings of anxiety and discouragement. Under such circumstances, achievement motivation as a component of self-regulation strategy can help undergraduates quickly adjust their outcome expectations to pursue success or to avoid failure, reversing their negative emotions and unemployment.

Self-regulation theory reflects the dynamic process by which people respond to discrepancies between actual performance and outcome expectations (Puranik et al., 2021). If the actual performance is higher than expected, individuals may actively pursue success by setting higher outcome expectations. When the actual performance is lower than outcome expectations, it is not a bad choice to avoid failure by lowering outcome expectations. It is important to note, however, that not all undergraduates will alter their job-seeking behaviors by adjusting their achievement motivation when the employment outcome is not satisfactory. For those who will not change their achievement motivation, strengthening the general self-efficacy in employment is also an effective strategy for teachers to enhance undergraduates’ employability. The study highlights the key role of achievement motivation in coping with various uncertain outcomes in the job-seeking process. It is conducive for teachers to deal with the problems of self-efficacy deficiency and expectation discrepancy in undergraduates’ job-hunting practice, thereby promoting the development of students’ employability.

### 4.4. The Mediating Role of Occupational Aspiration in the Relationship Between General Self-Efficacy and Employability

This study reveals that occupational aspiration plays a significant mediating role between general self-efficacy and employability, confirming Hypothesis 3. On one hand, general self-efficacy significantly and positively predicts occupational aspiration, consistent with previous research findings (Al-Bahrani et al., 2021). Self-efficacy is the basis of occupational aspiration. Individuals with higher self-efficacy are more willing to take on challenging tasks and set more ambitious career goals for themselves, thus having a higher level of occupational aspiration. On the other hand, occupational aspiration significantly and positively predicts employability, aligning with prior research findings (Mohammad, 2020). Occupational aspiration is the core component of the psychological motivation system, playing a crucial role in an individual’s employment performance. Occupational aspiration reflects an individual’s desire and determination to achieve success in a specific domain, which motivates individuals to acquire skills or develop abilities for successful employment. Additionally, the students who have higher occupational aspirations are more proactive in job-hunting, try more efficient job-searching strategies, make more detailed visionary career plans and are more persistent in adversity, which is conducive to acquiring higher employability. The study stresses the importance of occupational aspiration in improving employability, which accounts for why so many career guidance practices help students prepare for employment by setting long-term career goals or aspirations. Occupational aspiration encourages students to accumulate employment knowledge, skills and practical experience so as to obtain successful employment and develop sustainable employability.

### 4.5. The Chain Mediating Role of Achievement Motivation and Occupational Aspiration Between General Self-Efficacy and Employability

This study indicates that achievement motivation and occupational aspiration play a significant chain mediating role between general self-efficacy and employability, supporting Hypothesis 4. The chain mediating effect can be explained by employment practice and the Achievement Goal Theory.

In employment practice, achievement motivation and occupational aspiration both share a temporal component, as they are connected to future career expectations and beliefs. However, they operate at different stages of career development: occupational aspiration is directed to the long-term career goal, while achievement motivation is more closely linked to the short-term employment expectation in a specific situation. Students who report aspiration–expectation discrepancies will adjust both their aspirations and expectations in order to reach a dynamic balance between achievement motivation and occupational aspiration. The term employability is increasingly viewed as a person’s ability to obtain initial employment and maintain it or adapt to the rapid transitions between roles and environmental demands or even gain new employment if necessary (Lo Presti & Pluviano, 2016). It is inferred that employability involves getting a job and how to achieve better performance for sustainable development in the current protean career era. In other words, employability implies a chain effect of achievement motivation and occupational aspiration concerning a successive process: what to achieve and how to achieve better.

In addition, the Achievement Goal Theory proposes that achievement motivation triggers, guides and motivates individuals’ job-hunting behaviors. Individuals with high achievement motivation tend to favor careers that realize self-worth and expected occupational aspirations (Dweck & Leggett, 1988). Specifically, compared with individuals with low achievement motivation, those with high achievement motivation have higher levels of occupational aspiration and often prefer to pursue success-oriented “approach goals” rather than failure-avoidant “avoidance goals”. Research by Elliot and his colleagues (Elliot et al., 1999) confirms that setting challenging and achievable “approach goals” leads to better performance than setting “avoidance goals”. Thus, it is evident that achievement motivation and occupational aspiration play a chain mediating role between general self-efficacy and employability. Students with strong achievement motivation are more likely to choose challenging career goals, exhibit higher levels of occupational aspiration, participate in job-searching more actively and gain more opportunities for self-improvement, thus enhancing their employability.

In conclusion, general self-efficacy is an important antecedent affecting individuals’ choices of actions. Undergraduates with high general self-efficacy believe that they are capable of doing their jobs well. They hold more positive expectations for specific occupations or positions and thus form stronger achievement motivation. Undergraduates with stronger achievement motivation are more willing to take on challenging jobs and tend to set more ambitious career goals, thereby having a higher level of occupational aspiration. A higher level of occupational aspiration enables students to engage in job-seeking more proactively and effectively navigate setbacks and failures during the employment process, thereby enhancing their employability. It can be seen that general self-efficacy indirectly affects the employability of undergraduates through the chain mediating effects of achievement motivation and occupational aspirations. Therefore, to enhance undergraduates’ employability, it is necessary to focus on improving their general self-efficacy, achievement motivation and occupational aspiration.

## 5. Conclusions

Employability is a prerequisite for successful integration into the labor market. With the development of artificial intelligence technology, more and more employment uncertainties are emerging, which greatly reduces undergraduates’ self-efficacy of being employed in the protean career era, especially for undergraduates in regional universities with industrial features. The study, grounded in social cognitive career theory (SCCT), examines the impact of general self-efficacy on undergraduates’ employability, as well as the chain mediating roles of achievement motivation and occupational aspiration. A survey was conducted among 454 undergraduates from five regional universities with industrial features in Chongqing, utilizing the General Self-Efficacy Scale, Achievement Motivation Scale, Undergraduates’ Occupational Aspirations Questionnaire and Undergraduates’ Employability Questionnaire. The research findings were as follows:

(1) A significant positive correlation was found between general self-efficacy, achievement motivation, occupational aspiration and undergraduates’ employability.

(2) General self-efficacy was found to have a significant positive effect on undergraduates’ employability.

(3) General self-efficacy was found to influence undergraduates’ employability through three indirect pathways, namely the independent mediating effects of achievement motivation and occupational aspiration; and the chain mediating effects of achievement motivation and occupational aspiration.

### 5.1. Limitations and Future Directions

There are some limitations in the study. First, the measure of research variables is constrained in self-reported questionnaires. There may be subject bias because it is hard for students to perceive their psychological state with full accuracy. And the reliance on self-reported employability may be particularly sensitive to external shocks. For example, technological shocks like generative AI and robotics might both erode undergraduates’ self-efficacy and perceived employability, potentially driving the observed association. To address the issues, longitudinal design, integrating objective assessment to complement self-reported data or incorporating contextual moderators into the analytical model are worth considering in future research. Second, the participants in the study are all students from five regional universities with industrial features in Chongqing, China. A majority of participants are males and major in science and engineering, which reflects the demographic distribution in the industrial-featured universities in China. Future research should involve larger and more diverse samples to investigate whether the findings of the current study are still consistent. Third, the present study only examined the chain mediating role of achievement motivation and occupational ambition between general self-efficacy and undergraduates’ employability, and there may be other potential mediating variables. Future studies can investigate the mediating role of other individual or environmental variables between general self-efficacy and undergraduates’ employability. Moreover, the study does not consider how the emerging contextual factors impact the relationship between self-efficacy and employability. It is stated that students’ self-efficacy in employability is vulnerable to external inputs, such as exposure to AI-related discussions (Huseynov, 2025). Future research may reframe generative AI as a boundary condition to explore how emerging technologies, such as ChatGPT (GPT-3.5), impact the relationship between self-efficacy and employability.

### 5.2. Educational Implications

The findings of this study have important educational implications for interventions to improve the employability of undergraduates. Firstly, given that general self-efficacy has a significant positive effect on undergraduates’ employability, improving general self-efficacy is an effective strategy to enhance their employability. According to the Goal Setting Theory, when one has clear, challenging and achievable “high goals”, he will perform better. Therefore, guiding students to set clear and challenging employment goals will help undergraduates gain continuous affirmation during the job search process, thereby effectively enhancing their general self-efficacy. Secondly, considering that achievement motivation plays a mediating role between general self-efficacy and undergraduates’ employability, teachers should create a positive learning environment for students to stimulate their achievement motivation. Here are four tips for teachers to stimulate students’ achievement motivation: First, assign moderately difficult learning tasks for students to complete and offer more opportunities for students to experience success. Second, provide timely feedback to identify students’ strengths and potential, thereby stimulating their achievement motivation. Third, guide students to attribute positively, for example, attributing success or failure to controllable factors such as “the degree of persistence” or “the degree of making efforts”, which will help students engage in job hunting actively and ultimately be employed. Fourth, provide personalized assistance when students encounter setbacks during job searching, and social support from teachers will stimulate undergraduates’ achievement motivation. Finally, considering that general self-efficacy positively and significantly affects undergraduates’ employability through achievement motivation and occupational aspiration, higher education institutions should strengthen the training of career guidance teachers and motivate students to proactively plan their careers, set suitable and challenging occupational aspirations and deliberately shift occupational aspirations into real efforts to strengthen job readiness, thus enhancing their employability.

## Figures and Tables

**Table 1:** Analysis of variance in research variables across demographic characteristics (n = 454).

Characteristic Variables	Category	General Self-Efficacy	Achievement Motivation	Occupational Aspiration	Employability
		M ± SD	M ± SD	M ± SD	M ± SD
Gender	Male	2.40 ± 0.59	3.25 ± 0.62	3.31 ± 0.68	3.50 ± 0.72
	Female	2.34 ± 0.58	3.34 ± 0.59	3.38 ± 0.59	3.52 ± 0.60
	*t*	1.16	−1.40	−1.20	−0.31
Major	Non-STEM	2.21 ± 0.54	3.26 ± 0.66	3.25 ± 0.66	3.30 ± 0.69
	STEM	2.41 ± 0.59	3.28 ± 0.61	3.34 ± 0.65	3.54 ± 0.68
	*t*	−2.63 **	−0.29	−1.10	−2.67 **
Grade	Freshman	2.30 ± 0.65	3.18 ± 0.70	3.37 ± 0.79	3.52 ± 0.78
	Sophomore	2.37 ± 0.47	3.30 ± 0.60	3.27 ± 0.57	3.41 ± 0.66
	Junior	2.42 ± 0.66	3.40 ± 0.55	3.42 ± 0.61	3.53 ± 0.66
	Senior	2.44 ± 0.54	3.29 ± 0.57	3.28 ± 0.60	3.54 ± 0.62
	*F*	1.36	2.13	1.23	0.82
Origin	Rural	2.38 ± 0.57	3.31 ± 0.59	3.33 ± 0.68	3.47 ± 0.68
	County	2.33 ± 0.51	3.23 ± 0.59	3.27 ± 0.60	3.43 ± 0.70
	Urban	2.43 ± 0.67	3.27 ± 0.69	3.38 ± 0.66	3.63 ± 0.66
	*F*	0.97	0.62	0.88	3.07 *

Note: * *p* < 0.05, ** *p* < 0.01.

**Table 2:** Descriptive statistics and correlation analysis of research variables (n = 454).

Research Variables	M	SD	1	2	3	4
1 General Self-efficacy	2.38	0.58	1			
2 Achievement Motivation	3.28	0.62	0.23 **	1		
3 Occupational Aspiration	3.33	0.65	0.19 **	0.47 **	1	
4 Employability	3.50	0.68	0.37 **	0.32 **	0.47 **	1

Note: ** *p* < 0.01.

**Table 3:** Regression analysis of the relationship between variables in the chain mediation model (n = 454).

Model	Outcome	R	R^2^	*F*	β	*t*	LLCI	ULCI
Model 1								
①	②	0.294	0.086	2.961 ***	0.225	4.507 ***	0.127	0.323
Model 2								
①	③	0.518	0.269	10.730 ***	0.097	2.001 *	0.002	0.193
②					0.484	10.656 ***	0.395	0.573
Model 3								
①	④	0.624	0.390	17.453 ***	0.279	5.975 ***	0.187	0.371
②					0.100	2.055 *	0.004	0.196
③	0.518				0.364	7.958 ***	0.274	0.453

Note: To report the mediating effects more accurately, the data in Table 3 are presented with three decimal places. ① General self-efficacy, ② achievement motivation, ③ occupational aspiration and ④ employability. * *p* < 0.05, *** *p* < 0.001.

**Table 4:** Analysis of mediating effects of achievement motivation and occupational aspirations (n = 454).

Effect	Pathway	B	B%	95%CI	
				Lower	Upper
Total effect		0.376	100%	0.277	0.475
Direct effect		0.279	74.202%	0.187	0.371
Total indirect effect		0.097	25.798%	0.040	0.165
Indirect effect path1	①→②→④	0.023	6.117%	0.001	0.054
Indirect effect path2	①→③→④	0.035	9.309%	0.001	0.074
Indirect effect path3	①→②→③→④	0.040	10.638%	0.015	0.070

Note: ① General self-efficacy, ② achievement motivation, ③ occupational aspiration and ④ employability.

## Data Availability

The datasets generated and analyzed during the current study are available from the corresponding author upon reasonable request.
